# EsxA is required for antibacterial toxin export by the type VIIb secretion system

**DOI:** 10.1016/j.jbc.2026.113240

**Published:** 2026-06-09

**Authors:** Prakhar Y. Shah, Stephen R. Garrett, Timothy A. Klein, Roland Pfoh, Amogelang R. Raphenya, Andrew G. McArthur, P. Lynne Howell, John C. Whitney

**Affiliations:** 1 Michael DeGroote Institute for Infectious Disease Research, McMaster University, Hamilton, Ontario, Canada; 2 Department of Biochemistry and Biomedical Sciences, McMaster University, Hamilton, Ontario, Canada; 3 Program in Molecular Medicine, The Hospital for Sick Children, Toronto, Ontario, Canada; 4 David Braley Centre for Antibiotic Discovery, McMaster University, Hamilton, Ontario, Canada; 5 Department of Biochemistry, University of Toronto, Toronto, Ontario, Canada

**Keywords:** bacterial secretion systems, bacterial toxins, type VIIb secretion, protein export, protein-protein interactions, X-ray crystallography

## Abstract

Type VII secretion systems (T7SSb) are widespread in Bacillota and mediate both interbacterial antagonism and host interactions through the export of diverse effector proteins, including polymorphic Leu-X-Gly (LXG) toxins. Despite extensive variation in toxin repertoires, T7SSb systems universally secrete the small WXG100 protein EsxA, suggesting a conserved role in secretion. However, the relationship between EsxA and LXG toxin export remains unresolved. Here, we define the hierarchy and mechanistic basis of substrate export by the T7SSb of *Streptococcus intermedius*. We show that EsxA is required for the secretion of all LXG toxins, whereas EsxA export occurs independently of these effectors, establishing a unidirectional dependency. This requirement is not mediated by direct interaction, as EsxA does not associate with LXG toxin complexes. Instead, structural and mutational analyses reveal that EsxA forms a homodimer containing a bipartite export motif that is essential for both its own secretion and for LXG toxin export. Consistent with this, LXG toxins possess analogous composite export motifs, indicating that these substrates are independently recognized by the secretion machinery. We further show that while all four ATPase domains of the EssC secretion ATPase are required for system activity, only the Walker motifs of the D1 domain are necessary for substrate translocation. Finally, we demonstrate that EsxA export depends on EssC compatibility, indicating that EssC contributes to substrate recognition. Together, these findings establish EsxA as a conserved T7SSb substrate whose export is a prerequisite for LXG toxin secretion and define domain-specific requirements of EssC that underlie substrate recognition and translocation.

Type VII secretion systems (T7SS) are protein export pathways encoded by many Gram-positive bacteria (1, 2, 3, 4). These systems are broadly divided into two subtypes, T7SSa and T7SSb, which are found in Actinomycetota and Bacillota, respectively (2). In pathogenic mycobacteria, T7SSa systems are essential for virulence whereas in Bacillota, T7SSb systems have been implicated in both pathogenesis and interbacterial competition (5, 6, 7, 8, 9, 10, 11, 12, 13, 14, 15, 16, 17). Across both subtypes, the biological activities of the proteins secreted by these systems are thought to underlie their roles in microbial physiology and host interactions.

The best-studied T7SSb pathway is the ESS system of *Staphylococcus aureus* (18). This system secretes both WXG100 family proteins, such as EsxA, and larger antibacterial effectors, including the nuclease EsaD and the membrane-depolarizing toxin TspA (6, 7, 14, 19). Substrate export requires the activity of EssC, a membrane-embedded ATPase that forms the central channel of the secretion apparatus (20, 21). Comparative genomic studies of *S. aureus* strains have shown that the *essC* gene possesses a conserved 5′ region and a variable 3′ region, and that the latter determines which toxin substrates can be secreted by a given T7SSb (22). By contrast, EsxA secretion is conserved across EssC variants, suggesting that this substrate may play a central and universal role in ESS function (14, 22).

In streptococci, T7SSb systems export EsxA together with so-called polymorphic Leu-X-Gly (LXG) toxins, the latter of which mediate interbacterial antagonism and were first characterized in *Streptococcus intermedius* (6, 8, 14). In the absence of *essC*, LXG toxins are not secreted and T7SSb-dependent interbacterial antagonism is no longer observed (8). LXG toxins are typically encoded alongside small α-helical Lap targeting factors that form complexes with the LXG domain and are co-exported, providing the bipartite export signal required for secretion (5, 8, 23, 24, 25). Recent work further identified Ltc chaperones (formerly DUF4176 proteins) that, for a subset of LXG toxins, bind to an intrinsically disordered region, stabilize the toxin in a secretion-competent state, and are retained in the cytoplasm following toxin export (23, 26, 27). Collectively, these studies establish the composition of LXG toxin complexes. However, the secretion hierarchy between EsxA and the complexes formed by LXG toxins and their associated Lap targeting factors remains unclear.

In mycobacterial T7SSa systems, variation in the C-terminal ATPase domain of EccC has been linked to substrate specificity, with distinct alleles promoting the secretion of different effector subsets (28, 29). By contrast, the principles of substrate recognition and translocation remain poorly defined for T7SSb. Although LXG toxins require Lap targeting factors and, in some cases, Ltc chaperones for stability and secretion, how these accessory proteins intersect with EssC function is not understood. The contribution of EsxA is also unresolved; while secretion of EsxA is universally conserved, its relationship to the export of other substrates remains unclear (14, 30). Together, these uncertainties represent knowledge gaps in our understanding of substrate hierarchy and the role of EssC’s ATPase domains in T7SSb export.

Here, we use *S*. *intermedius* as a model to define how EsxA and EssC contribute to substrate export by the T7SSb. We show that LXG toxins require EsxA for secretion, but that EsxA export occurs independently of these effectors. Structural and mutational analyses reveal that EsxA forms a homodimer with a bipartite export motif that is essential for its own secretion and for LXG toxin export. Additionally, we demonstrate that while all four ATPase domains of EssC are necessary for T7SSb activity, only the Walker motifs of the D1 ATPase domain are required for substrate translocation. Finally, by comparing *S. intermedius* strains, we show that divergent EssC and EsxA homologs are not functionally interchangeable. Together, these findings establish EsxA as a conserved T7SSb substrate whose export is a prerequisite for LXG toxin secretion and uncover domain-specific requirements of EssC that underlie T7SSb function.

## Results

### LXG toxin export requires *esxA*

**Figure 1:**
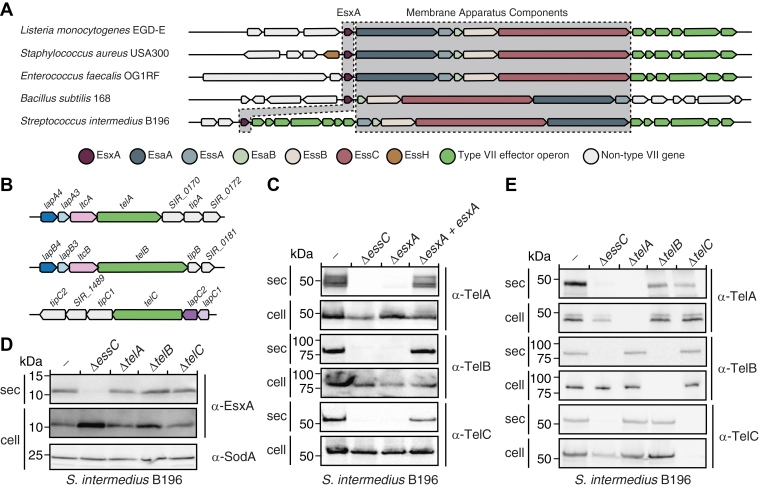
**EsxA is required for LXG toxin secretion but not *vice versa*.***A*, genomic organization of T7SSb loci in representative Bacillota, showing conserved *esxA* and membrane apparatus genes flanked by variable regions. *B*, genomic organization of LXG toxin loci in *S. intermedius* B196 (*Si*^B196^), including genes encoding LXG toxins (*green*), cognate immunity proteins (Tip; *gray*), Ltc chaperones (*pink*), and Lap targeting factors (*blue, light blue, purple, light purple*). *C*, deletion of *esxA* abolishes secretion of all three LXG toxins, which is restored upon complementation. Immunoblot analysis of TelA, TelB, and TelC in cell and supernatant fractions from WT *Si*^B196^, the indicated deletion mutants, and complemented strains. *D*, EsxA secretion is unaffected by loss of LXG toxins. Immunoblot analysis of EsxA in WT *Si*^B196^, a secretion-deficient control (Δ*essC*), and strains lacking individual LXG toxins (Δ*telA*, Δ*telB*, Δ*telC*). *E*, deletion of any single LXG toxin does not affect secretion of the others. Immunoblot analysis of TelA, TelB, and TelC in strains lacking individual LXG toxins. LXG, Leu-X-Gly.

The genes encoding EsxA and the T7SSb apparatus are conserved across the phylum Bacillota (Fig. 1*A*). Additionally, clusters of T7SSb genes that vary at the species or even strain level are found in all characterized T7SSb-containing bacteria, and these genes often flank the conserved apparatus genes (Fig. 1*A*). Several recent studies have shown that T7SSb exported LXG toxins, along with their co-exported targeting factors, cytoplasmic chaperones, and cognate immunity proteins are encoded within these variable gene clusters (6, 8, 24, 26). For example, our model T7SSb bacterium *S*. *intermedius* B196 (*Si*^B196^) possesses two LXG toxin gene clusters on either side of its T7SSb locus, encoding the TelA and TelB toxins, respectively, and a third gene cluster located elsewhere on the chromosome encoding the TelC toxin (Fig. 1, *A* and *B*). These observations, combined with available extracellular proteomics data for *Si*^B196^, indicate that most T7SSb systems likely export a single EsxA protein and a variable number of LXG toxins (8). However, the functional relationship between these two families of T7SSb exported proteins has yet to be examined.

Given the conservation of *esxA* among T7SSb-containing bacteria, we posited that this gene is likely required for the secretion of the Tel toxins by the T7SSb of *Si*^B196^. To test this, we used an *esxA* mutant and examined the ability of this strain to export each of the Tel toxins using Western blot analysis of *Si*^B196^ cell and supernatant fractions. To control for spontaneous cell lysis, we included a Δ*essC* strain because the absence of the gene is known to disrupt the secretion of all T7SSb substrates (8). This analysis revealed that in the absence of *esxA*, none of the Tel toxins are secreted from the cell (Fig. 1*C*). Importantly, this loss of toxin export is not due to polar effects on the downstream T7SSb apparatus genes caused by deleting *esxA* because Tel toxin secretion is restored when EsxA is expressed from a plasmid.

We next wanted to determine if EsxA is similarly reliant on any of the Tel toxins for its T7SSb-dependent export. Therefore, we next generated mutants lacking *telA*, *telB*, or *telC* and assessed EsxA secretion in each background. For each of these mutant strains, we found that EsxA is both produced and exported at levels comparable to WT *Si*^B196^ (Fig. 1*D*). Thus, the dependency of LXG toxin secretion on *esxA* is not bidirectional. Finally, we asked if any secretion interdependency exists between members of the LXG toxin family. Using the same toxin deletion strains described above, we observed no changes in the overall Tel toxin secretion profile when any single Tel toxin encoding gene is absent (Fig. 1*E*). Collectively, these results demonstrate that LXG toxin export in *Si*^B196^ is unidirectionally dependent on EsxA.

### EsxA does not interact with LXG toxin complexes

**Figure 2:**
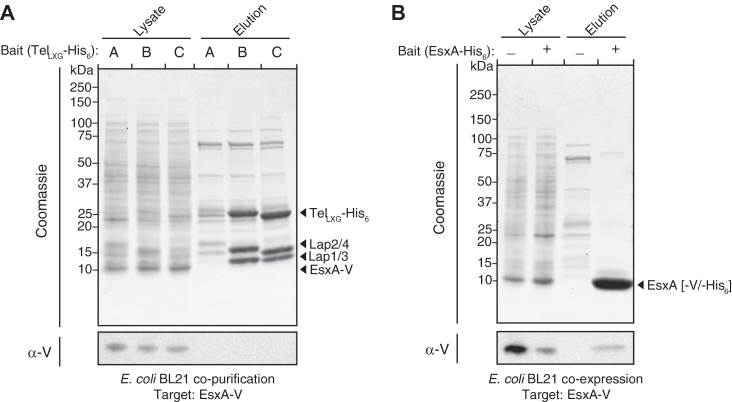
**EsxA does not interact with LXG toxin complexes.***A*, copurification of VSV-G-tagged EsxA with His_6_-tagged LXG domains from TelA, TelB, and TelC (expressed with their cognate Lap proteins) following nickel affinity purification. *B*, EsxA self-associates. Coexpression and copurification of VSV-G- and His_6_-tagged EsxA following nickel affinity purification. LXG, Leu-X-Gly.

We previously showed that the C-terminal toxin domains of TelA and TelC are dispensable for their secretion (23). Therefore, we reasoned that if the dependency on *esxA* for Tel toxin export is due to a physical interaction between the proteins, this interaction would occur between EsxA and the N-terminal LXG domains of TelA, TelB, and TelC. To test for a direct interaction between EsxA and LXG domains, we performed a series of pairwise co-purification experiments using His_6_-tagged LXG domains from TelA, TelB, and TelC and VSV-G (V) epitope-tagged EsxA. We previously found that the LXG domains of TelA, TelB, and TelC form heterotrimeric complexes with cognate Lap proteins for stability and export (23). Untagged Lap proteins were therefore included to recapitulate the known LXG-Lap interactions required for complex formation. Despite all proteins expressing to high levels in a heterologous system, EsxA-V did not co-purify with any of the LXG domain-containing heterotrimeric complexes (Fig. 2*A*). To ensure that EsxA-V was properly folded when expressed in *Escherichia. coli*, we took advantage of the observation that EsxA proteins from other T7SSb containing bacteria have been shown to dimerize (31, 32). By co-expressing EsxA-V with His_6_-tagged EsxA, we observed that EsxA-V co-purifies with EsxA-His_6_ after nickel affinity chromatography indicating that *Si*^B196^ EsxA is likely also dimeric and therefore in its native folded state (Fig. 2*B*). Thus, our data indicate that under these experimental conditions, which are conducive to LXG-Lap heterotrimer formation, there is no evidence of a direct interaction between EsxA and the Tel toxins.

### EsxA is a homodimeric protein with a conserved C-terminal export motif

Given EsxA does not directly interact with the Tel proteins, we wanted to further understand its function in T7 secretion and to do so, we first determined its three-dimensional structure by X-ray crystallography. EsxA was expressed and purified from *E. coli* (Fig. S1*A*), and we obtained crystals that diffracted to 1.48 Å. The crystallographic phase problem was overcome using an *ab initio* phasing approach that uses an α-helical fragment search with the ARCIMBOLDO_LITE binary (33). The final model of EsxA was refined to an *R*_work_/*R*_free_ of 20.7% and 22.7%, respectively (Table 1).

**Table 1:** X-ray data collection and refinement statistics

	EsxA
Data Collection	
Beamline	NSLSII 17-ID-1
Wavelength (Å)	0.92009
Space group	C2
Cell dimensions	
*a, b, c* (Å)	63.463, 22.549, 61.300
*a, b, g* (°)	90.00, 102.16, 90.00
Resolution^a^ (Å)	25.45–1.48 (1.53–1.48)
Unique reflections	14,382 (1362)
CC_1/2_^b^	99.7 (0.95)
*R*_merge_^c^	7.3 (63.3)
*R*_pim_^b^	3.1 (28.0)
*I*/σ*I*	14.73 (2.31)
Completeness (%)	99.2 (93.5)
Redundancy	6.4 (6.2)
Wilson B-factor	16.8
Refinement	
*R*_work_^d^/*R*_free_ (%)	20.7/22.7
Average B-factors (Å^2^)	
Protein	19.5
Water	30.1
No. atoms	
Protein	742
Water	84
Rms deviations	
Bond lengths (Å)	0.004
Bond angles (°)	0.6
Ramachandran plot^e^ (%)	
outliers/allowed/favored	0/0/100
Coordinate error (Å)	0.26
PDB code	12FA

a Values in parentheses correspond to the highest resolution shell.

b As defined by Karplus and Diederichs (78).

c *R*_merge_ = Σ_h_Σ_j_|I_hj_–<I_h_>|/Σ_h_Σ_j_I_hj_, where I_hj_ is the intensity of observation j of reflection h.

d *R* = Σ_h_|F_o_|–|F_c_|/Σ_h_|F_o_| for all reflections, where F_o_ and F_c_ are observed and calculated structure factors, respectively. *R*_free_ is calculated analogously for the test reflections, randomly selected, and excluded from the refinement.

e As defined by MolProbity (79).

**Figure 3:**
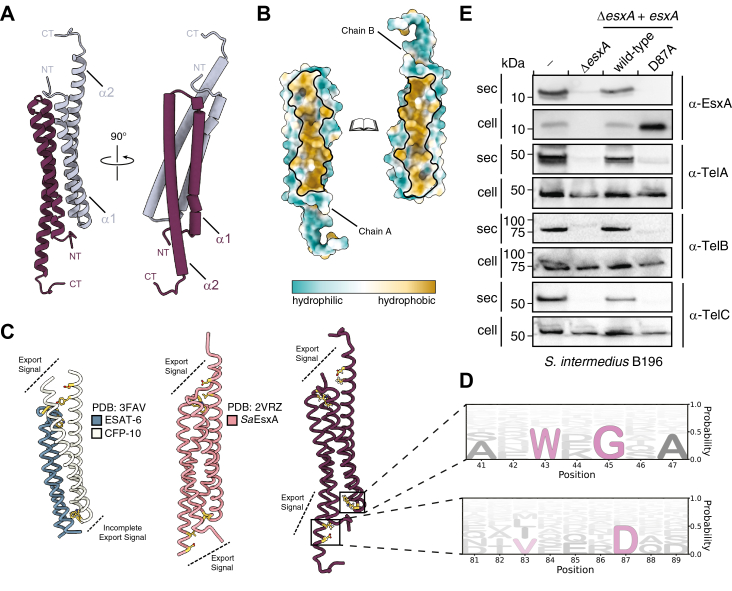
**EsxA forms a homodimer with a bipartite export motif required for secretion.***A*, crystal structure of the *Si*^B196^ EsxA homodimer shown in two orientations (90° rotation). Secondary structure elements and N- and C-termini are indicated for each protomer. *B*, surface representation of the EsxA homodimer highlighting the hydrophobic dimer interface. Surface coloring corresponds to hydrophilicity (*blue*) and hydrophobicity (*yellow*). *C*, structural comparison of *Si*^B196^ EsxA (*purple*) with homodimeric *S. aureus* EsxA (PDB: 2VRZ; *pink* (32)) and the heterodimeric *M. tuberculosis* EsxA-EsxB complex (PDB: 3FAV; teal and white (31)). Bipartite export motifs are indicated. *D*, sequence conservation of the WxG and ΦxxxD motifs shown as sequence logos. Conserved residues are highlighted. *E*, mutation of the conserved C-terminal acidic residue (D87A) abolishes secretion of EsxA and LXG toxins. Immunoblot analysis of EsxA and TelA-C in cell and supernatant fractions from strains expressing WT or mutant EsxA. LXG, Leu-X-Gly.

The overall structure of EsxA reveals a head-to-tail homodimer that forms a α-helical bundle with approximate dimensions of ∼100 Å by ∼25 Å by ∼25 Å (Fig. 3*A*). Within this bundle, each EsxA molecule adopts a helix-turn-helix topology with an approximate 30° offset angle between the two protomers. The buried surface area between the interacting EsxA protomers measures 1528 Å^2^, and an analysis of the surface properties of this interface indicates that it is almost exclusively hydrophobic in nature, suggesting that homodimer formation is highly favorable in aqueous environments and largely an entropy-driven process (Fig. 3*B*). Consistent with this prediction, analysis of EsxA’s solution state molecular weight using size exclusion chromatography coupled with multi-angle light scattering yielded a mass of 18.8 kDa, indicating that the protein is also homodimeric in solution (Fig. S1*B*). This is consistent with findings for homologs of this protein in other bacteria and further supports the conclusion that EsxA proteins have a strong propensity to form dimers (31, 32). Taken together, these observations suggest that EsxA exists as a homodimer under physiological conditions.

Despite only having 39% and 20% pairwise sequence identity, respectively, the *Si*^B196^ EsxA homodimer closely resembles that of *S*. *aureus* EsxA homodimers (PDB: 2VRZ (32)) and *Mycobacterium tuberculosis* EsxA:EsxB heterodimers (PDB: 3FAV (31)) with a Cα RMSD of 2.4 Å and 2.0 Å, respectively (Fig. S1*C*). Homodimeric *S. aureus* EsxA and heterodimeric *M. tuberculosis* EsxA:EsxB both possess a bipartite export sequence and in the latter case this sequence is known to be required for T7SS-dependent export from the cell (34). This export sequence consists of a WxG motif contributed by the turn region of EsxA and a conserved acidic residue near the C-terminus of EsxB (Fig. 3*C*) (31, 34). For homodimeric EsxA proteins, this structural arrangement results in the formation of two pseudosymmetric export sequences that are related by a 180° rotation (31, 32). *Si*^B196^ EsxA possesses both conserved motifs with its WxG motif being comprised of Trp43 and Gly45 whereas Asp87 is the conserved acidic residue, which is part of a loosely conserved ‘VxxxD’ motif (Fig. 3*D*).

To probe the functional importance of EsxA’s putative export sequence, we introduced a D87A mutation into our *esxA* complementation plasmid and tested the ability of this variant to support T7SSb-dependent protein export in our *Si*^B196^ Δ*esxA* strain. Despite showing no differences in stability or overall secondary structure compared to the WT protein, EsxA^D87A^ was not secreted by the T7SSb of *Si*^B196^ and instead accumulated in the cytoplasm (Figs. 3*E* and S1, *A*, *B*, *D*). Furthermore, the export of TelA, TelB, and TelC is lost in this strain indicating that the export motif of EsxA not only governs its own secretion but also the secretion of *Si*^B196^’s LXG toxin repertoire.

### EsxA represents a minimal T7SSb export domain

The finding that EsxA does not interact with the Tel toxins is somewhat unexpected, as a direct interaction coupled with the presence of an export signal would provide a plausible mechanism for EsxA-dependent recruitment of Tel toxins to the T7SSb apparatus. An alternative possibility is that LXG toxins encode their own export signals and are therefore independently recognized by the secretion machinery, with EsxA fulfilling a separate role in the export process. To explore this possibility, we examined the crystal structures of previously characterized substrates from both T7SSa and T7SSb systems.

The structure of EsxA closely resembles a portion of the LXG domain and the LapA3 component of the recently solved TelA_LXG_-LapA3-LapA4 co-crystal structure (23). In this comparison, EsxA aligns well with the TelA_LXG_-LapA3 portion of the complex despite accounting for only ∼50% of its total volume (Fig. S2). Notably, TelA_LXG_ and LapA3 form the same composite WxG and ΦxxxD secretion signal observed in EsxA. Similar export signals are also present in T7SSa substrates, including PE–PPE heterodimers and EspB (Fig. S2) (35, 36, 37, 38). Taken together, these observations suggest that LXG toxins, together with one of their cognate Lap proteins, form export motifs that are chemically indistinguishable from that of EsxA. This provides a structural explanation for why direct protein-protein interactions between EsxA and the Tel toxins are not required for their secretion.

### All four ATPase domains of EssC are required for T7SSb substrate export

Having demonstrated that Tel substrates have their own export motif, we next explored the possibility that EsxA is required for their export at the point of recruitment to the secretion apparatus. In well-studied T7SSa systems, the membrane-embedded EccC ATPase adopts a hexameric arrangement that forms a substrate translocation pore (39, 40, 41). These structural studies further show that a large cytoplasmic region of EccC contains four FtsK/SpoIIIE-family AAA+ ATPase domains. Structural analysis of EssC, the T7SSb homolog of EccC, suggests it may play a similar role (21, 42, 43). The ATPase domains of EccC/EssC have been implicated in recruiting T7SS substrates to the translocation pore (43, 44)); however, the functional contributions of the individual EssC ATPase domains to T7SSb substrate export remain poorly understood.

**Figure 4:**
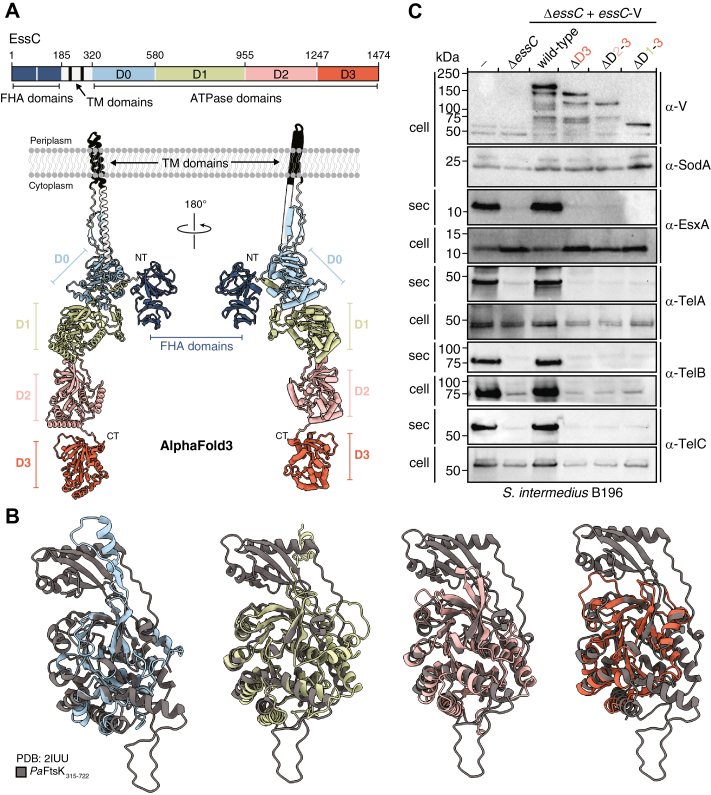
**All four EssC ATPase domains are required for T7SSb substrate export.***A*, domain architecture of *Si*^B196^ EssC and corresponding AlphaFold3 structural model. Amino acid positions are indicated, and domains are color-coded as follows: forkhead-associated (FHA; *blue*), transmembrane regions (*TM; black*), and ATPase domains D0-D3 (*light blue, green, pink, and orange, respectively*). B) Structural alignment of EssC ATPase domains (D0-D3) with the FtsK ATPase domain (PDB: 2IUU; *gray* (45)), showing conservation of the Rossmann fold. Cα RMSD values for D0-D3 are 2.9 Å, 2.8 Å, 2.6 Å, and 2.8 Å, respectively. *C*, truncation of any ATPase domain abolishes secretion. Immunoblot analysis of EsxA and TelA-C in cell and supernatant fractions from Δ*essC* strains expressing VSV-G-tagged WT or C-terminally truncated EssC variants. Superoxide dismutase (SodA) serves as a loading control, and VSV-G detection confirms protein expression.

Consistent with prior studies of EssC, AlphaFold3 confidently predicts that *Si*^B196^ EssC is composed of two FHA domains, two TMDs, and four ATPase domains (Figs. 4*A* and S3*A*). These ATPase domains, named D0, D1, D2, and D3, adopt a typical Rossmann fold and align well with the characterized ATPase domain of FtsK, from which the EssC-containing superfamily of ATPases derives its name (Figs. 4*B* and S3*B*) (42, 45, 46). To begin to understand substrate recruitment to the T7SSb complex, we first asked whether each ATPase domain is required for substrate export. To test this, we expressed VSV-G (V)-tagged WT EssC or truncation variants lacking one, two, or three ATPase domains in the *Si*^B196^ Δ*essC* strain and assessed EsxA and Tel toxin secretion. As expected, expression of WT EssC-V restored secretion of all T7SSb substrates, whereas expression of any EssC truncation rendered the T7SS nonfunctional (Figs. 4*C* and S3*C*–*F*). This observation corroborates evidence from the T7SSb of *S. aureus*, where each EssC ATPase domain is required to maintain an intact membrane apparatus (42, 43, 44).

### Functional Walker motifs in D1 are necessary for substrate export

**Figure 5:**
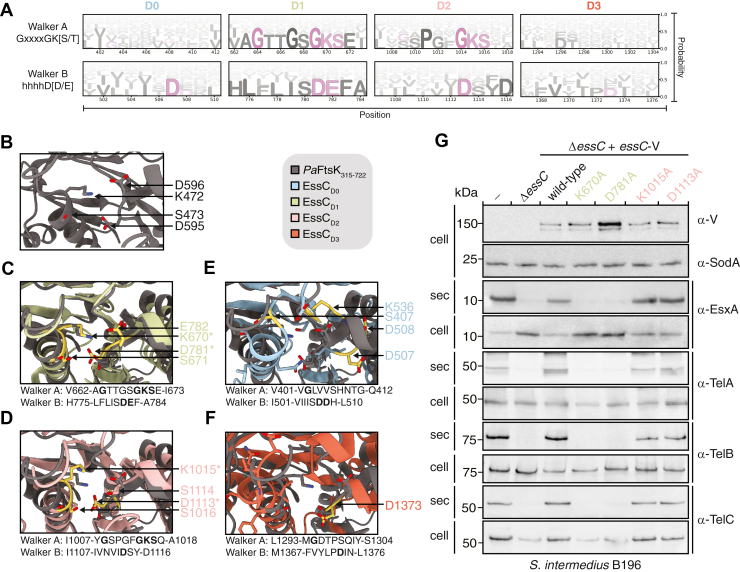
**Walker motifs in the EssC D1 domain are required for substrate export.***A*, sequence conservation of Walker A and Walker B motifs across EssC ATPase domains (D0-D3). Conserved residues are highlighted, with the most conserved positions indicated in *pink*. *B*, structural representation of residues involved in ATP binding and hydrolysis in FtsK. Key residues (K472, S473, D595, D596) are highlighted in *yellow* to indicate their established roles in catalysis. *C*–*F*, structural alignment of EssC ATPase domains with FtsK (PDB: 2IUU; *gray* (45)). Individual domains are shown as follows: (*C*) D0 (*light blue*), (*D*) D1 (*green*), (*E*) D2 (*pink*), and (*F*) D3 (*orange*). Residues corresponding to Walker motifs or predicted catalytic positions are highlighted in *yellow* in the structural models and indicated in bold in the aligned sequences. *G*, mutations in the Walker A or Walker B motifs of the D1 domain abolish secretion. Immunoblot analysis of EsxA and TelA-C in cell and supernatant fractions from Δ*essC* strains expressing VSV-G-tagged WT or mutant EssC variants. SodA serves as a loading control, and VSV-G detection confirms protein expression.

Because deletion of individual EssC ATPase domains did not reveal a specific site of substrate recruitment, we next took a more targeted approach to examine the role of ATP binding and hydrolysis in T7SSb function. Walker A and Walker B motifs are essential for ATP binding and hydrolysis across the AAA+ ATPase superfamily (47). Walker A is defined by a ‘GxxxxGK[S/T]’ motif, whereas Walker B consists of a ‘hhhhD[D/E]’ motif, where ‘h’ represents a hydrophobic amino acid. A multiple sequence alignment of EssC homologs together with *Pseudomonas aeruginosa* FtsK were used to identify Walker A and Walker B motifs in the D0, D1, D2, and D3 domains of EssC. This analysis revealed a canonical Walker A motif in D1 and a partially conserved motif in D2 (Fig. 5*A*). By contrast, no recognizable Walker A motif was identified in either D0 or D3. Similarly, a well-conserved Walker B motif is present in D1, whereas only a single conserved acidic residue was detected within the expected Walker B regions of D0 and D2 (Fig. 5*A*).

The ATPase domain of the FtsK DNA translocase has been extensively characterized and serves as a reference for the AAA+ ATPase superfamily. In *P. aeruginosa* FtsK, residues K472 and S473 of the Walker A motif are required for ATP binding, whereas residues D595 and D596 of the Walker B motif mediate ATP hydrolysis (Fig. 5*B*) (45, 47). Mutation of any of these residues abolishes FtsK activity. Structural comparison of the FtsK ATPase domain with our AlphaFold3 model of EssC revealed that the intact and partial Walker motifs in the D1 and D2 domains align well with those of FtsK. In EssC, the D1 Walker A and B motifs correspond to K670/S671 and D781/D782, respectively, whereas the partially conserved D2 motifs correspond to K1015/S1016 and D1113/S1114 (Fig. 5, *C* and *D*).

Although our sequence analysis did not identify Walker motifs in D0 and D3, we examined structural alignments to determine whether alternative residues might support ATP binding or hydrolysis. In D0, residues D507 and D508 lie near the position expected for a Walker B motif; however, their side chain orientations do not align with the catalytic residues of FtsK and are not positioned to support ATP hydrolysis (Fig. 5*E*). Similarly, structural comparison with FtsK did not reveal candidate residues that could substitute for Walker A or Walker B motifs in D3 (Fig. 5*F*).

To test whether functional ATPase domains are required for substrate export by EssC, we mutated the lysine in the Walker A motif and the first aspartate in the Walker B motif of both D1 and D2, and expressed these EssC variants from a plasmid in the *Si*^B196^ Δ*essC* strain. While EssC was expressed in all strains, export of EsxA and TelA-C was abolished when either the Walker A or Walker B motifs of D1 were mutated (Figs. 5*G* and S4, *A*–*D*). In contrast, inactivating mutations in the Walker motifs of D2 had no effect on substrate export (Figs. 5*G* and S4, *A*–*D*). Together, these data demonstrate that the Walker A and Walker B motifs of the D1 domain, but not D2, are required for the export of T7SSb substrates, consistent with prior studies showing that mutations in catalytic residues of EccC/EssC impair substrate export (43, 44, 46, 48).

### EsxA export depends on compatibility with EssC

**Figure 6:**
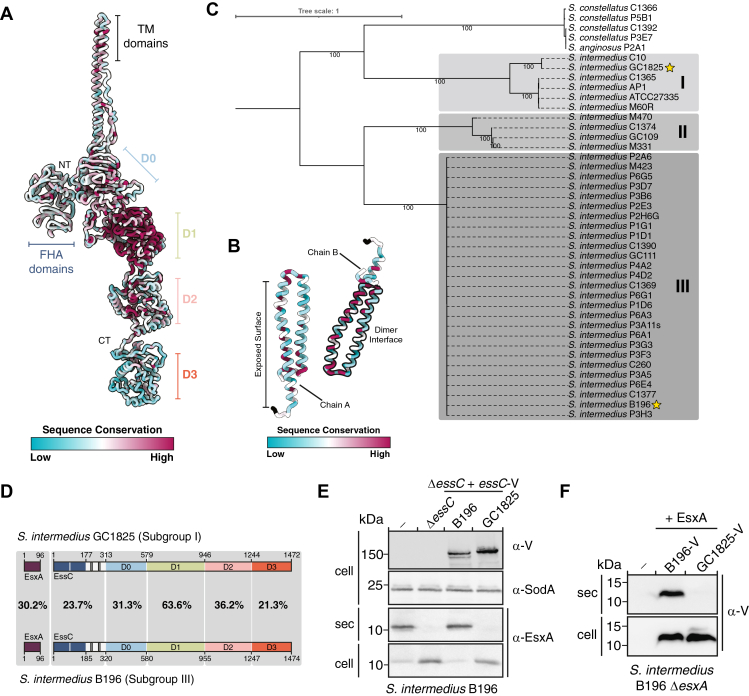
**EsxA secretion depends on compatibility with EssC.***A*, sequence conservation mapped onto the AlphaFold3 model of EssC. Conservation is shown on a blue (*low*) to maroon (*high*) scale. *B*, sequence conservation mapped onto the crystal structure of EsxA. Regions of interest, including the exposed surface and dimer interface, are indicated. Conservation is shown on a *blue (low) to maroon (high)* scale. *C*) Maximum likelihood phylogeny of EssC homologs from the *Streptococcus anginosus* group, showing clustering into subgroups I (*light gray*), II (*gray*), and III (*dark gray*). Model strains *S. intermedius* B196 and GC1825 are indicated by *yellow stars*. Branch lengths are shown except for dashed branches (unresolved polytomies). Numbers represent bootstrap values for specific nodes. *D*, domain architecture of EssC from *S. intermedius* B196 and GC1825. Amino acid positions are indicated, and domains are color-coded as follows: forkhead-associated (FHA; *blue*), transmembrane regions (TM; *black*), and ATPase domains D0-D3 (*light blue, green, pink, and orange*, respectively). Percent sequence identity between corresponding domains is indicated. *E*, non-native EssC does not restore EsxA secretion. Immunoblot analysis of EsxA in cell and supernatant fractions from *Si*^B196^ Δ*essC* strains expressing VSV-G-tagged EssC from either *S. intermedius* B196 or GC1825. SodA serves as a loading control, and VSV-G detection confirms protein expression. *F*, EsxA secretion requires a compatible EssC homolog. Immunoblot analysis of EsxA-V from either *S. intermedius* B196 or GC1825 expressed in a *Si*^B196^ Δ*esxA* strain.

Given the requirement for functional Walker motifs in the D1 domain of EssC for T7SSb substrate export, we sought to identify the role this might play in substrate recognition. A multiple sequence alignment of EssC homologs from strains within the *Streptococcus anginosus* group revealed substantial sequence divergence across the protein, with strong conservation observed primarily within the D1 ATPase domain (Fig. 6*A*). Interestingly, EsxA homologs encoded by these strains are also highly divergent (Fig. 6*B*). To better understand this diversity, we constructed a maximum likelihood phylogeny of EssC homologs from the *S. anginosus* group. While EssC proteins from *S. anginosus* and *Streptococcus constellatus* cluster together, *S. intermedius* encodes three distinct EssC subgroups (Fig. 6*C*). We found that EssC from our model strain *S. intermedius* B196 belongs to subgroup III, whereas a second strain used in our laboratory, *S. intermedius* GC1825, belongs to subgroup I (Fig. 6*C*). Given the sequence divergence between EssC proteins from these strains, we used them to test whether EsxA export depends on compatibility between divergent EssC and EsxA homologs.

We found that EssC from *Si*^B196^ and *Si*^GC1825^ share relatively low sequence identity (21–37%) across most of the protein, with the notable exception of the D1 ATPase domain, which shares 63.6% identity (Fig. 6*D*). EsxA homologs from these strains are similarly divergent, sharing only 30.2% sequence identity (Fig. 6*D*). Given the sequence divergence of both proteins, we asked whether EssC from one strain could complement secretion in the other. To test this, we expressed *Si*^GC1825^ EssC in a *Si*^B196^ Δ*essC* strain and examined EsxA secretion. Although *Si*^GC1825^ EssC was expressed, it failed to restore secretion of EsxA in *Si*^B196^ (Fig. 6*E*). To determine whether EssC from *Si*^B196^ could secrete a heterologous EsxA homolog, we next expressed either *Si*^B196^ EsxA or *S. intermedius* GC1825 EsxA in a *Si*^B196^ Δ*esxA* strain and assessed secretion. While both proteins were expressed, only *Si*^B196^ EsxA was secreted by the *Si*^B196^ T7SSb apparatus (Fig. 6*F*).

Together, these findings indicate that EsxA export in *Si*^B196^ requires compatibility between EsxA and EssC homologs. Despite substantial divergence between EssC proteins from *Si*^B196^ and *Si*^GC1825^, the D1 ATPase domain remains conserved, consistent with its essential catalytic role in T7SSb substrate export. However, the inability of either EssC or EsxA homologs to function across strains indicates that diversification of EssC proteins likely contributes to substrate recognition within *S. intermedius* T7SSb systems.

## Discussion

The T7SSb exports proteins through a membrane-embedded apparatus formed by the AAA+ ATPase EssC, but the principles governing substrate recruitment and export hierarchy remain poorly defined. Here, we identify a functional relationship between EsxA and LXG toxin export in *S*. *intermedius* in which toxin secretion depends on EsxA, whereas EsxA export proceeds independently of these substrates. This asymmetry indicates that EsxA function precedes LXG toxin secretion and is required to enable LXG toxin export by the T7SSb. Notably, this requirement is not explained by direct association because EsxA does not detectably interact with LXG toxin complexes, arguing against a role as a substrate-specific adaptor and instead indicating that EsxA acts at the level of the T7SSb secretion apparatus.

Previous work in *S. aureus* showed that deletion of *esxA* abolishes secretion of the WXG100 proteins EsxB and EsxC whereas loss of *esxB* or *esxC* does not impair EsxA export (30). Although initially interpreted as evidence for a secretion hierarchy among WXG100 substrates, subsequent studies demonstrated that EsxB and EsxC form a complex with the LXG-like nuclease toxin EsaD and are required for its export (25). These findings indicate that apparent dependencies can arise from obligate substrate complexes rather than ordered secretion. In contrast, our data show that EsxA-dependent toxin export occurs without detectable interaction between EsxA and LXG toxin complexes, supporting a distinct mechanism in which EsxA functions independently of toxin-associated targeting factors. Together, these observations suggest that a requirement for EsxA export may represent a conserved feature of T7SSb systems.

Our structural and mutational analyses further support a model in which EsxA functions as a minimal export-competent substrate. The EsxA homodimer contains a bipartite export motif that is essential for its secretion and, indirectly, for LXG toxin export. At the same time, LXG toxins, together with their cognate Lap targeting factors, encode analogous composite motifs that recapitulate this architecture (23). These observations suggest that T7SSb substrates share a common structural solution for recognition by the secretion machinery, regardless of whether the motif is encoded within a single polypeptide or assembled from multiple components. In this context, the requirement for EsxA is unlikely to reflect a role in substrate recruitment through direct binding. Instead, EsxA export may be necessary to establish or maintain a secretion-competent state of the apparatus that permits subsequent translocation of larger substrates. Consistent with this interpretation, disruption of EssC function abolishes secretion of both EsxA and LXG toxins. Truncation of any of the four ATPase domains renders the system inactive, in agreement with observations in *S. aureus* where deletion of the D3 domain also eliminates secretion (43, 44). However, this contribution does not appear to involve ATP hydrolysis for three of these domains. Only the Walker A and Walker B motifs of the D1 domain are required for substrate export, whereas analogous mutations in D2 do not impair secretion. This contrasts with findings in *S. aureus*, where mutations in the C-terminal ATPase domains disrupt export (43, 44), suggesting that the contributions of these domains may vary across organisms. Together, these data support a model in which D1 is the primary ATPase domain required for translocation, while the remaining domains contribute structurally to assembly or stability of the secretion apparatus.

The role of EssC in substrate recognition has been proposed to reside within its C-terminal ATPase domains, with D1 implicated in recognition of EsxA and D2–D3 proposed to engage LXG–Lap substrate complexes. This model is based largely on studies in *S. aureus*, where EssC variants with divergent C-terminal regions retained the ability to secrete EsxA but no other substrates (20, 22). In those systems, both EssC D1 and EsxA are highly conserved, complicating interpretation of substrate specificity. In contrast, the EssC and EsxA homologs examined here are substantially diverged, and we find that divergent EssC homologs fail to support EsxA export across strains. These findings indicate that compatibility between EssC and EsxA is required for secretion and suggest that substrate recognition cannot be attributed solely to highly conserved regions of the ATPase. While D1 remains essential for activity, the inability of divergent homologs to function interchangeably supports a model in which multiple regions of EssC contribute to substrate discrimination.

Together, our findings support a model in which EsxA represents a conserved, minimal substrate whose export is required to enable secretion by the T7SSb whereas LXG toxins are independently recognized through analogous export motifs. In this model, EsxA does not function as a direct adaptor for toxin recruitment but instead acts upstream of substrate translocation, likely by modulating the activity or conformation of the secretion apparatus. These results define a functional hierarchy among T7SSb substrates and explain how diverse effectors are accommodated by a conserved export pathway.

## Experimental procedures

### Bacterial strains, plasmids, and growth conditions

*E. coli* was grown in lysogeny broth at 37 °C at 225 revolutions per minute under aerobic conditions, supplemented with 50 μg/ml of kanamycin, 150 μg/ml of carbenicillin, or 100 μg/ml spectinomycin where required. *E. coli* XL1-Blue and BL21 (DE3) CodonPlus were used for molecular cloning and protein overexpression, respectively. *S. intermedius* was grown in Todd Hewitt broth supplemented with 0.5% yeast extract (THY) in a 37 °C stationary 5% CO_2_ incubator. All *S. intermedius* strains were grown on THY agar plates for 1 to 3 days prior to growth in THY broth to ensure uniform growth rate. THY broth and agar plates were supplemented with 250 μg/ml of kanamycin or 75 μg/ml of spectinomycin to select for gene deletions and pDL277-derived plasmids, respectively. The complete list of bacterial strains used in this study is listed in Table S1. When cloning into pDL277, the P96 promoter sequence from *Streptococcus pneumoniae* was fused upstream to the gene of interest (49). A comprehensive list of the plasmids used in this study is found in Table S2.

### DNA manipulation

To prepare genomic DNA, *S. intermedius* overnight cultures were pelleted and resuspended in 50 μl PBS pH 7.4, then mixed in a 1:1 ratio with InstaGene Matrix (BioRad) after which the extraction protocol was followed, as per manufacturer guidance. Oligonucleotides used in this study were generated by Integrated DNA Technology. Molecular cloning was performed using Phusion polymerase, restriction endonucleases, and T4 DNA ligase from NEB. Sanger sequencing was performed by The Center for Applied Genomics at The Hospital for Sick Children and whole plasmid sequencing was performed by Plasmidsaurus.

### Transformation of *S. intermedius*

Linear DNA fragments for allelic replacement (see below) or plasmids were transformed into *S. intermedius* as previously described (50). In brief, *S. intermedius* overnight cultures were diluted 1:10 into 2 ml THY broth supplemented with 5 μl of 0.1 mg/ml *S. intermedius* competence stimulating peptide (DSRIRMGFDFSKLFGK, synthesized by Genscript) and incubated for 2 h at the appropriate growth conditions. Between 100 to 200 ng of linear or plasmid DNA was then added, and cultures were grown for an additional 3 h before plating on THY agar plates supplemented with the appropriate antibiotic.

### Gene deletion in *S. intermedius* by allelic exchange

*S. intermedius* gene deletions were performed as described previously (51). In short, gene deletion constructs were assembled in pETDuet-1 plasmid. These constructs were comprised of 1000 bp upstream of the gene of interest (5′ flank) including the first 15 to 45 bp of the gene of interest, a spectinomycin promoter derived from pDL277, a kanamycin resistance cassette from pBAV1K, and 1000 bp downstream of the gene of interest (3′ flank) including the last 15 to 45 bp of the gene of interest (52). These deletion constructs were subsequently digested from the plasmid, gel extracted (Monarch DNA GEL Extraction Kit, NEB) and added to competence peptide stimulated *S. intermedius* cells.

### Secretion assays

Culture tubes containing 2 ml THY broth were inoculated with *S. intermedius* colonies from THY agar plates and grown overnight (final OD_600_ of 1.0–1.1). Overnight cultures were then diluted 1:100 into 20 ml THY and grown overnight for a second iteration. Cell and secreted (sec) fractions were then separated by centrifugation at 4000 *g* for 15 min. The cell samples were washed once in 1 ml of PBS pH 7.4 and centrifuged at 4000 *g* for 5 min. PBS wash was decanted, and the cell samples were resuspended in 150 μl of 1:1 ratio of PBS to 4× Laemmli buffer, after which the cell samples were boiled for 10 min. For the sec samples 10% trichloroacetic acid was added and the samples were incubated at 4°C overnight to precipitate secreted protein. The sec samples were then centrifuged at 35,000 *g* for 30 min and resulting pellets were washed once with 20 ml of 95% cold acetone. Sec samples were centrifuged again at 35,000 *g* for 30 min and the acetone was removed. The sec samples were allowed to air dry briefly on ice before the precipitated protein pellets were resuspended in 100 μl of 1:2 ratio of 4× Laemmli buffer to 8M urea and boiled for 10 min. Cell and sec samples were analyzed for proteins of interest by SDS-PAGE and western blotting.

### SDS-PAGE, gel electrophoresis, western blotting and protein staining

A tris-tricine buffer (200 mM Tris, 100 mM Tricine, 0.1% SDS, pH 8.3) was used for all SDS-PAGE gels. Gels were run at 85 V for 15 min then at 170 V for 35 min. Proteins were visualized using Coomassie stain (30% methanol, 10% glacial acetic acid, 0.1% w/v Coomassie brilliant blue R-250). Gels were rinsed in deionized water, incubated with Coomassie for 20 min, destained in 30% methanol, 10% glacial acetic acid for 3 h and imaged using a ChemiDoc XRS + imaging system (Bio-Rad). For western blotting, gels were transferred to nitrocellulose at 100 V for 30 min (α-TelA-D and α-VSV-G (α-V)) or to methanol-activated PVDF at 80 V for 1 h (α-EsxA). The blots were blocked with 5% skim milk in TBS-T for 30 min before being incubated with primary antibody for 1 h (1:3000 for α-V and α-TelC, or 1:5000 for α-EsxA and α-TelA,B,D). Blots were washed three times with 15 ml of TBS-T and then incubated with goat α-rabbit secondary antibody (1:5000) for 45 min. Blots were again washed three times in TBS-T before being developed with Clarity Max Western ECL reagent and imaged on a ChemiDoc XRS+ (Bio-Rad).

### Protein expression, purification and pulldowns

WT and mutant EsxA were expressed in BL21 (DE3) CodonPlus. The strains were diluted 1:50 in LB supplemented with 50 μg/ml of kanamycin and grown to an OD_600_ 0.50 to 0.55. Protein expression was then induced by the addition of 1 mM IPTG and protein was expressed overnight (∼18 h) at 18 °C. The cells were pelleted, resuspended in lysis buffer (20 mM Tris-HCl pH 8.0, 300 mM NaCl, 10 mM imidazole), and sonicated four times for 30 s at 30% amplitude. Cellular debris was cleared by 30 min of centrifugation at 35,000 *g*. Lysate was passed over Ni-NTA resin and washed three times with 20 ml of lysis buffer. Protein was eluted with 4 ml of elution buffer (20 mM Tris-HCl pH 8.0, 300 mM NaCl, 400 mM imidazole). For protein crystallization trials and downstream biophysical analysis, the eluants were then further purified by size exclusion chromatography using a HiLoad 16/600 Superdex 200 or Superdex 200 Increase 10/300 Gl columns on an ÄKTA explorer (Cytiva). A similar protocol was used for protein pulldowns with noted exceptions. VSV-G tagged EsxA was grown in LB supplemented with 150 μg/ml of carbenicillin while His tagged Tel_LXG_-Lap-Lap complexes were grown in LB supplemented with 50 μg/ml of kanamycin and 150 μg/ml of carbenicillin. These strains were then co-lysed and then co-purified as described above.

### Antibody generation

A custom polyclonal antibody for TelB was generated for this study. Because full-length TelB is toxic to *E. coli*, overexpression of just the LXG domain of TelB (TelB_LXG_) was performed for antibody production (8).To improve TelB_LXG_ stability, we coexpressed and copurified TelB_LXG_ with LapB3 and LapB4. This complex was purified in PBS pH 7.4 and yielded 10 mg of total protein that was subsequently shipped to Genscript for custom antibody production. The generation of the α-EsxA, α-TelA, α-TelC, α-TelD and α- superoxide dismutase A antibodies was described previously (8, 23, 24, 51).

### Protein crystallization

WT EsxA at 10 mg/ml was screened for crystallization conditions using the hanging drop vapor diffusion method and the MCSG suite of sparse matrix crystal screens (Anatrace). Crystals formed after one to 2 weeks in 0.1 M Bis-Tris:HCl pH 5.5, and 2 M ammonium sulfate. The crystals were cryoprotected using the same buffer supplemented with 25% ethylene glycol and flash frozen in liquid nitrogen prior to X-ray data collection.

### Protein structure prediction, visualization, and analysis

Protein secondary structure predictions were generated by PSIPRED 4.0 on the UCL PSIPRED Workbench at http://bioinf.cs.ucl.ac.uk/psipred/ (53). Protein structure predictions were performed with AlphaFold3 available to the public at https://golgi.sandbox.google.com/ (54). Predicted models were manually assessed and selected based on the per-residue pLDDT and pAE scores and the biological plausibility of the predicted domains in the polypeptide. UCSF ChimeraX was used for protein structure analysis and figure generationavai (55). Surface hydrophobicity calculations were performed using default parameters (56). Interchain interfaces were analyzed using PDBePISA accessible at https://www.ebi.ac.uk/msd-srv/prot_int/cgi-bin/piserver (57). All structural alignments and reported RMSD scores were calculated by the DALI webserver using PDB search and Pairwise alignment tools available at http://ekhidna2.biocenter.helsinki.fi/dali/ (58). Volume analysis was done using USCF ChimeraX (https://www.rbvi.ucsf.edu/chimera) default tools.

### X-ray data collection, structure determination, and model refinement

Data collection was performed using the Brookhaven National Laboratory 17-ID-1 beamline at the National Synchrotron Light Source II. Diffraction data were collected at 100 K with 0.5 s exposure and a 0.2 degree of rotation for a total of 360 degrees. The crystal diffracted to 1.48 Å and diffraction images were collected on a Pixel Dectris Eiger X 16M detector at 150 mm distance using an X-ray wavelength of 13.475 keV (0.92009 Å). Diffraction data was integrated with XDS and processed using CCP4 program suite (59, 60, 61). The structure of EsxA was solved using Arcimboldo_Lite which combines a fragment search by Phaser with density modification and autotracing by Shelxe (33, 62, 63). The program automatically built most backbone residues of the two long α-helices of EsxA. Coot was used to model side chains, the connecting loop and the termini while computational structural refinement was performed with Phenix.refine until the *R*_work_ and *R*_free_ converged to 20.7% and 22.7%, respectively (64, 65). Collection and refinement statistics for EsxA can be found in Table 1 and the structure is deposited in the Protein Data Bank under PDB: 12FA.

### Size exclusion chromatography coupled with multi-angle light scattering analysis of protein complexes

Size exclusion chromatography with multi-angle laser light scattering was performed on WT and mutant EsxA. The proteins were expressed and purified as described above and concentrated to 2 mg/ml by spin filtration. Size exclusion chromatography was run on a Superdex 200 column (GE Healthcare), and multi-angle laser light scattering was conducted using a MiniDAWN and Optilab system (Wyatt Technologies). Data was collected and analyzed using the Astra software (https://www.wyatt.com/products/software/astra.html) package (Wyatt Technologies), ava.

### Circular dichroism spectroscopy

Circular dichroism spectroscopy was performed on WT and mutant EsxA. The proteins were expressed and purified as described above and concentrated to 0.1 mg/ml by spin filtration. The experiment was performed on a JASCO J-1100 instrument and data were collected and analyzed using the CDPro software (https://jascoinc.com/products/spectroscopy/circular-dichroism/software/applications-software) package (JASCO).

### Sequence analysis, maximum likelihood trees and sequence logo generation

To identify homologous sequences to EsxA and EssC from *S. anginosus* group genomes, Parnsp 1.7.4 was first used to removal clonal genomes with 100% identity (66). Nonclonal genome taxonomy was predicted using GTDB-Tk 1.7.0 and gene calling was performed using Prodigal 2.6.3 (67, 68). Hmmscan 3.3.1 was used to predict protein domains (69). Protein sequences were aligned using MUSCLE version 5.1 with default parameters (70), regions of uncertain homology were removed using trimAl (71), and maximum likelihood trees generated with RAxML 8.2.12 using the PROTCATIJTTF substitution model (72). HMMs for each protein sequence alignment was generated using the Skylign (https://skylign.org) webserver, set to “create HMM – remove mostly empty columns” (73). The resulting matrices were downloaded as tabular text, formatted, and then visualized using Logomaker (74).

### Western blot quantification

For semi-quantitative analysis of protein secretion, Western blot band intensities from cell and supernatant fractions were quantified using Fiji v2.9.0 (75). TIFF images were converted to 8 bit grayscale and mean gray values were measured using a uniform region of interest applied across all bands within a blot. To account for uneven background signal, local background measurements were obtained for each lane from nearby regions lacking detectable protein signal. Signal intensities were inverted using the formula 255 − X, where X represents the measured mean gray value, and background-corrected values were calculated by subtracting local background measurements. Relative secretion was determined by calculating the ratio of secreted (“Sec”) to cellular (“Cell”) signal intensity.

## Data availability

All data are contained within this article and the supporting information file. The atomic coordinates and structure factors for the EsxA structure were deposited into the Protein Data Bank under the accession number PDB: 12FA.

## Supporting information

This article contains supporting information (8, 23, 24, 31, 32, 37, 38, 45, 58, 76, 77).

## Conflict of interest

The authors declare that they have no conflicts of interest with the contents of this article.
