# Supplementary material for: EsxA is required for antibacterial toxin export by the type VIIb secretion system
Source: J Biol Chem. 2026 Jun 9;302(7):113240. doi: 10.1016/j.jbc.2026.113240 (PMC13355648; doi:10.1016/j.jbc.2026.113240)
Supplement: Supporting Figures and Tables [file mmc1.docx]

**Supporting information**

**EsxA is required for antibacterial toxin export by the type VIIb secretion system**

Prakhar Y. Shah*^1,2^, Stephen R. Garrett*^1,2^, Timothy A. Klein^1,2^, Roland Pfoh^3^, Amogelang R. Raphenya^1,2^, Andrew G. McArthur^1,2,4^, P. Lynne Howell^3,4^, and John C. Whitney^1,2,4,*^

^1^Michael DeGroote Institute for Infectious Disease Research, McMaster University, Hamilton, ON, L8S 4K1, Canada

^2^Department of Biochemistry and Biomedical Sciences, McMaster University, Hamilton, ON, L8S 4K1, Canada

^3^Program in Molecular Medicine, The Hospital for Sick Children, Toronto, ON, M5G 0A4, Canada

^4^David Braley Centre for Antibiotic Discovery, McMaster University, Hamilton, ON, L8S 4K1, Canada

^5^Department of Biochemistry, University of Toronto, Toronto, ON, M5S 1A8, Canada

Running title: Effector secretion hierarchy of the T7SSb

Keywords: type VII secretion system (T7SS), protein export, bacterial toxin, *Streptococcus*, ATPase, substrate specificity.

*These authors contributed equally.

^ To whom correspondence should be addressed: John C. Whitney, Department of Biochemistry and Biomedical Sciences, McMaster University, Hamilton, Ontario, L8S 4K1, Canada.

E-mail: [jwhitney@mcmaster.ca](mailto:jwhitney@mcmaster.ca).

**
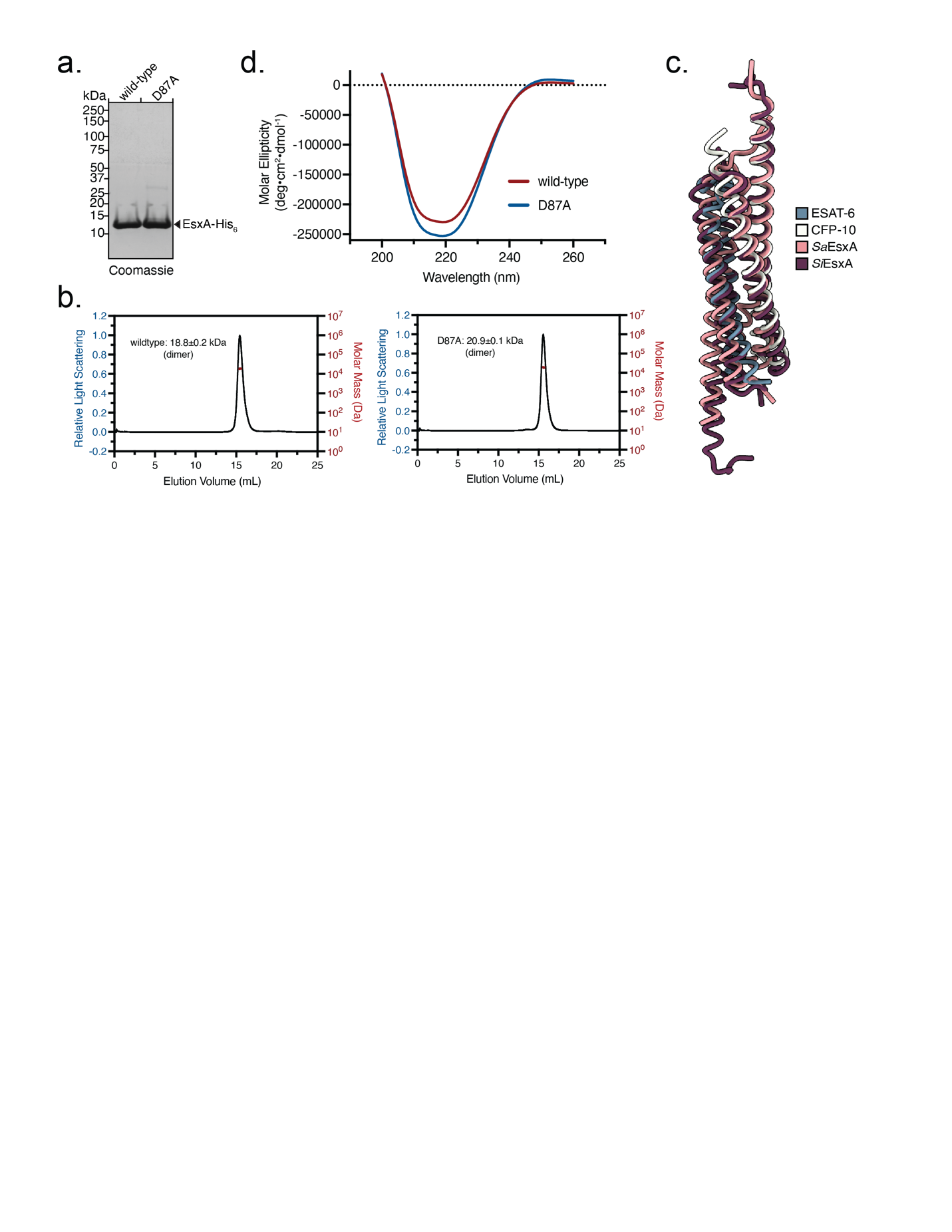
**

**Fig. S1. Wild-type and D87A EsxA form homodimers with similar structural properties.** A) Purified EsxA analyzed by SDS-PAGE following size exclusion chromatography (SEC). B) SEC-MALS analysis of purified wild-type and D87A EsxA. In both cases, the measured molecular weight is consistent with a homodimer. C) Structural comparisons of EsxA homologs. Pairwise structural alignments of the ESAT-6/CFP-10 heterodimer (PDB: 3FAV (1)), *S. aureus* EsxA (PDB: 2VRZ (2)), and S*. intermedius* B196 EsxA were performed using the DALI server (3). D) The D87A mutation does not alter secondary structure. Circular dichroism spectroscopy of wild-type and D87A EsxA shows comparable spectra, indicating similar secondary structure content.

**
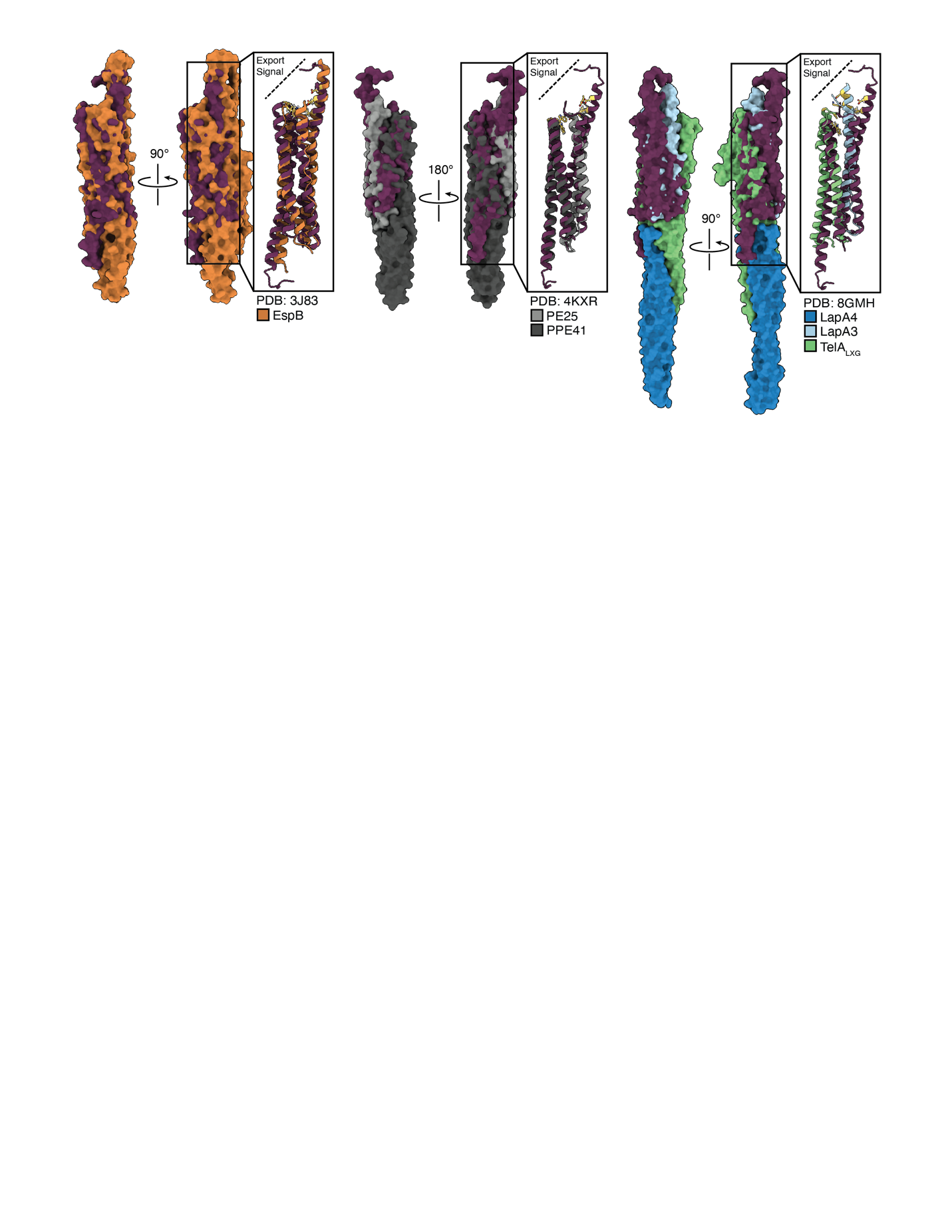
**

**Figure S2. EsxA represents a minimal T7SS export module.** Structural overlays of the EsxA homodimer (purple) with EspB (PDB: 3J83; orange (4)), PE25-PPE41 (PDB: 4KXR; black and grey (5)), and the TelA_LXG_-Lap complex (PDB: 8GMH; blue, light blue, and green (6)). Expanded views highlight the conserved structural features associated with T7SS export, including the α-helical bundle and bipartite export motif composed of a ‘WxG’ (or equivalent) sequence and a conserved acidic residue (both shown in yellow).

**
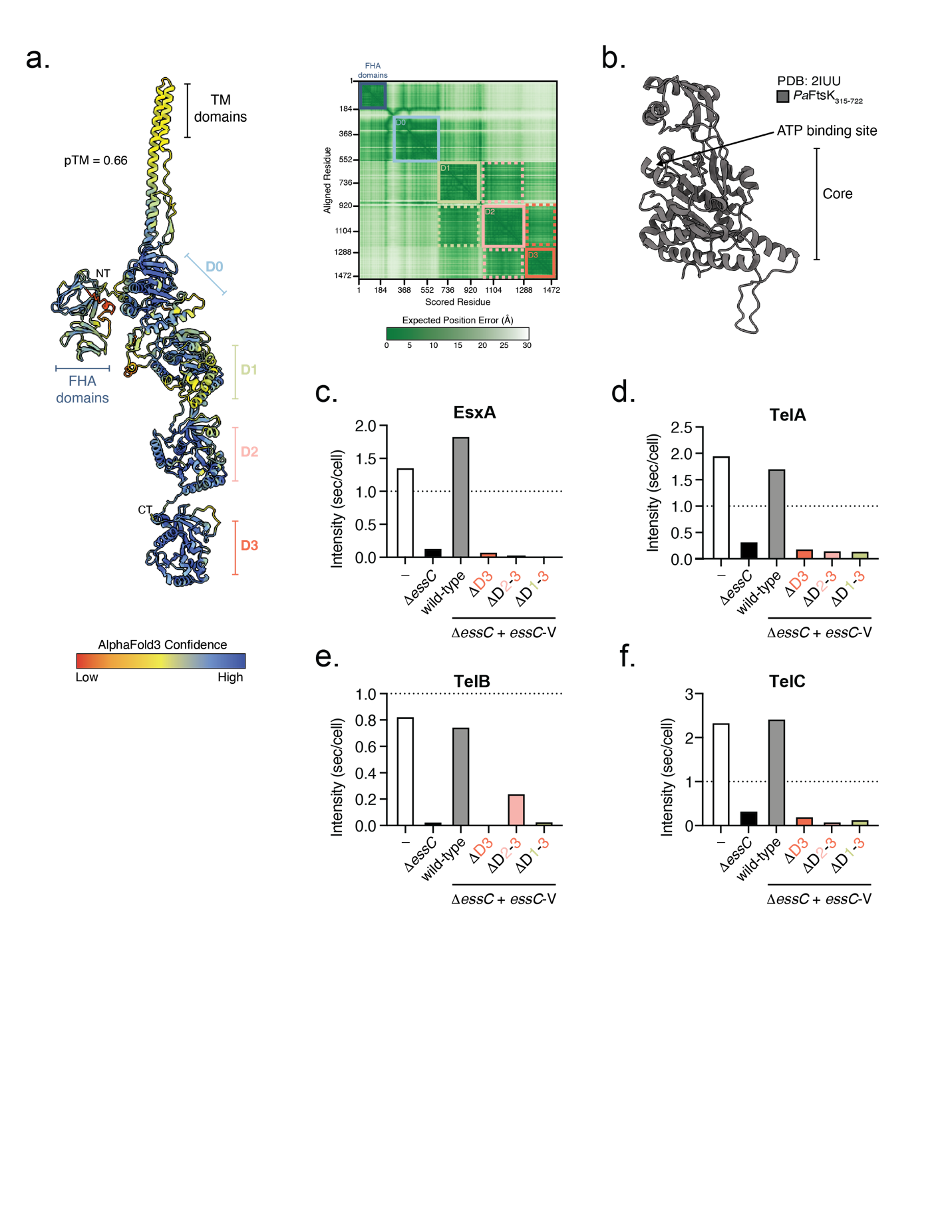
**

**Fig. S3. Domain analysis of EssC and its role in secretion.** A) AlphaFold3 structural model of EssC from *S. intermedius* B196 with annotated domains. Model confidence is shown on a scale from red (low) to blue (high). The predicted aligned error (PAE) plot is shown from dark green (low error) to light green (high error). EssC domains are highlighted using the same colour scheme as in Figure 4. B) Structure of FtsK highlighting regions relevant to this study, including the ATP-binding site and ATPase core (PDB: 2IUU; grey (7)). C-F) Densitometric quantification of the western blots presented in Fig. 4C showing secretion of C) EsxA, D) TelA, E) TelB, and F) TelC in the indicated EssC mutant backgrounds. Secreted protein levels were normalized to the corresponding cellular signal.

**
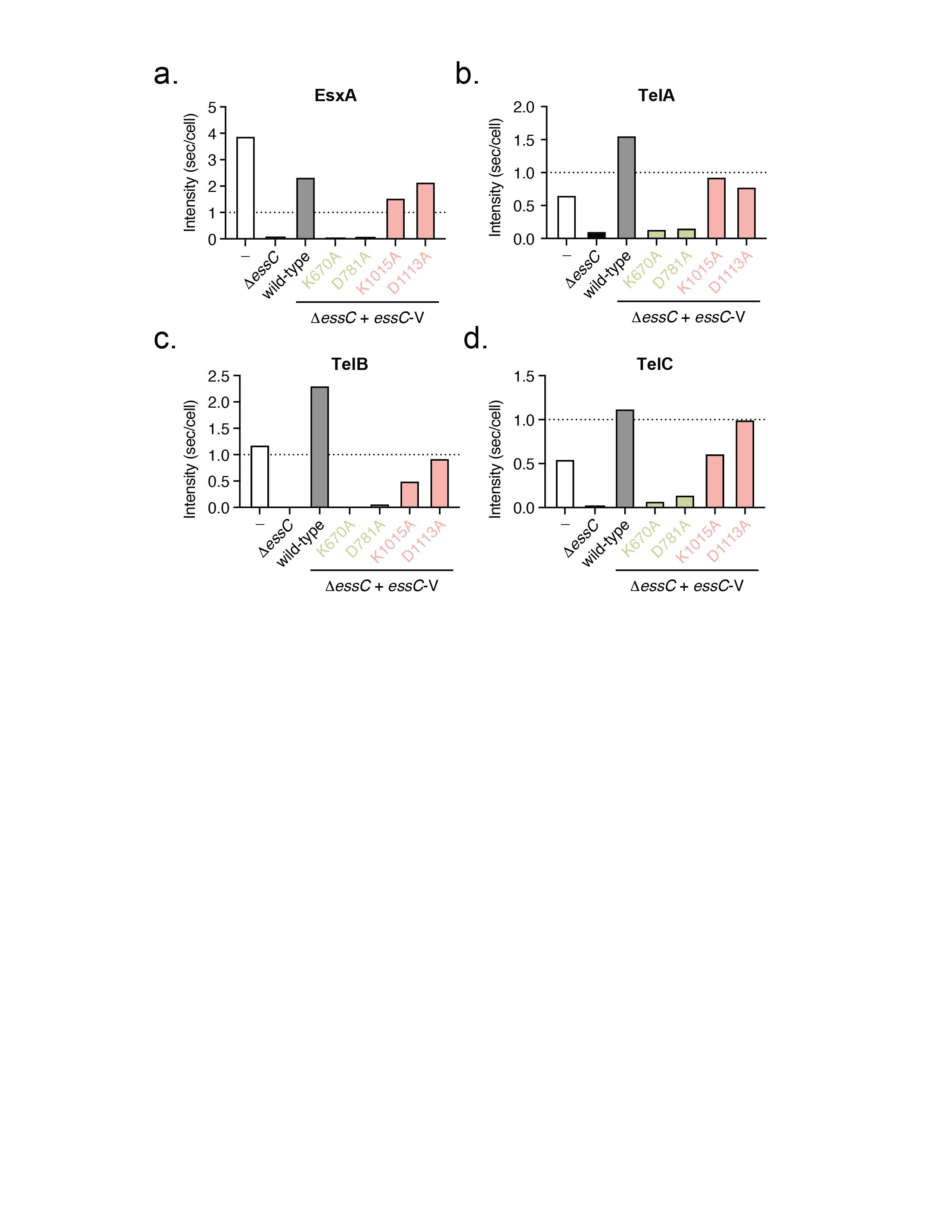
**

**Fig. S4. Semi-quantitative analysis of protein secretion by EssC Walker mutants.** A-D) Densitometric quantification of the western blots presented in Fig. 5G showing secretion of A) EsxA, B) TelA, C) TelB, and D) TelC in the indicated EssC mutant backgrounds. Secreted protein levels were normalized to the corresponding cellular signal.

**Table S1: Strains used in this study.**

| **Organism** | **Genotype** | **Description** | **Reference** |
| --- | --- | --- | --- |
| *S. intermedius* B196 | Wildtype |  | (8) |
|  | ΔSIR_0166::kan^R^ | *esxA* deletion strain | This study |
|  | ΔSIR_0169::kan^R^ | *telA* deletion strain | This study |
|  | ΔSIR_0175::kan^R^ | *essC* deletion strain | (9) |
|  | ΔSIR_0179::kan^R^ | *telB* deletion strain | This study |
|  | ΔSIR_1489::kan^R^ | *telC* deletion strain | This study |
| *E. coli* XL-1 Blue | *recA1 endA1 gyrA96 thi-1 hsdR17 supE44 relA1 lac* [*F’ proAB lacI^q^* Z Δ M15 Tn*10* (Tet^R^)] | Cloning strain | Agilent |
| *E. coli* BL21 (DE3) CodonPlus | F^-^ *ompT gal dcm lon hsdS*_B_(r_B_^-^ m_B_^-^) λ(DE3) pLysS(Cm^R^) | Protein expression strain | Novagen |

**Table S2: Plasmids used in this study.**

| **Plasmid** | **Relevant features** | **Reference** |
| --- | --- | --- |
| pDL277 | *Streptococcus*-*E. coli* shuttle vector, Spec^R^ | (10) |
| pDL277::P96_*esxA* | *S. intermedius* expression vector for B196 EsxA | This study |
| pDL277::P96_*esxA_*D87A | *S. intermedius* expression vector for B196 EsxA D87A mutation | This study |
| pDL277::P96_*esxA*_VSV-G | *S. intermedius* expression vector for B196 EsxA, C-terminal VSV-G tag | This study |
| pDL277::P96_*essC_*VSV-G | *S. intermedius* expression vector for B196 EssC, C-terminal VSV-G tag | This study |
| pDL277::P96_*essC_*ΔD3_VSV-G | *S. intermedius* expression vector for B196 EssC C-terminal truncation of D3, C-terminal VSV-G tag | This study |
| pDL277::P96_*essC_*ΔD2-3_VSV-G | *S. intermedius* expression vector for B196 EssC C-terminal truncation of D2-3, C-terminal VSV-G tag | This study |
| pDL277::P96_*essC_*ΔD1-3_VSV-G | *S. intermedius* expression vector for B196 EssC C-terminal truncation of D1-3, C-terminal VSV-G tag | This study |
| pDL277::P96_*essC_*K670A_VSV-G | *S. intermedius* expression vector for B196 EssC K670A mutation, C-terminal VSV-G tag | This study |
| pDL277::P96_*essC_*D781A_VSV-G | *S. intermedius* expression vector for B196 EssC D781A mutation, C-terminal VSV-G tag | This study |
| pDL277::P96_*essC_*K1015A_VSV-G | *S. intermedius* expression vector for B196 EssC K1015A mutation, C-terminal VSV-G tag | This study |
| pDL277::P96_*essC_*D1113A_VSV-G | *S. intermedius* expression vector for B196 EssC D1113A mutation, C-terminal VSV-G tag | This study |
| pDL277::P96_*esxA*_GC1825_VSV-G | *S. intermedius* expression vector for EsxA from *S. intermedius* GC1825, C-terminal VSV-G tag | This study |
| pDL277::P96_*essC*_GC1825_VSV-G | *S. intermedius* expression vector for EssC from *S. intermedius* GC1825, C-terminal VSV-G tag | This study |
| pETDuet-1 | Co-expression vector with *lacI*, T7 promoter, N-terminal His_6_ tag in MCS1, Amp^R^ | Novagen |
| pETduet-1::5′-*esxA*_flank_SpecPromoter_kanR_3′- *esxA*_flank::empty | Plasmid containing *S. intermedius* B196 *esxA* knockout construct for allelic exchange | This study |
| pETduet-1::5′-*telA*_flank_SpecPromoter_kanR_3′- *telA*_flank::empty | Plasmid containing *S. intermedius* B196 *telA* knockout construct for allelic exchange | This study |
| pETduet-1::5′-*telB*_flank_SpecPromoter_kanR_3′- *telB*_flank::empty | Plasmid containing *S. intermedius* B196 *telB* knockout construct for allelic exchange | This study |
| pETduet-1::5′-*telC*_flank_SpecPromoter_kanR_3′- *telC*_flank::empty | Plasmid containing *S. intermedius* B196 *telC* knockout construct for allelic exchange | This study |
| pETDuet-1::*esxA*_VSV-G::empty | *E. coli* co-expression vector for the B196 EsxA, C-terminal VSV-G tag | This study |
| pETDuet-1::*telA*_LXG__His_6_::*lapA4* | *E. coli* co-expression vector for the B196 TelA LXG domain with LapA4, C-terminal His_6_ on TelA | (6) |
| pETDuet-1::*telB*_LXG__His_6_::*lapB4* | *E. coli* co-expression vector for the B196 TelB LXG domain with LapB4, C-terminal His_6_ on TelB | (6) |
| pETDuet-1::*telC*_LXG__His_6_::*lapC2* | *E. coli* co-expression vector for the B196 TelC LXG domain with LapC2, C-terminal His_6_ on TelC | (11) |
| pET29b | Expression vector with *lacI*, T7 promoter, C-terminal His_6_ tag, Kan^R^ | Novagen |
| pET29b::*esxA*_His_6_ | *E. coli* expression vector for B196 EsxA, C-terminal His_6_ | This study |
| pET29b::*lapA3* | *E. coli* expression vector for B196 LapA3 | (6) |
| pET29b::*lapB3* | *E. coli* expression vector for B196 LapB3 | (6) |
| pET29b::*lapC1* | *E. coli* expression vector for B196 LapC1 | (11) |

**References**

1. Poulsen, C., Panjikar, S., Holton, S. J., Wilmanns, M., andSong, Y. H. (2014) WXG100 protein superfamily consists of three subfamilies and exhibits an alpha-helical C-terminal conserved residue pattern PLoS One **9**, e89313 10.1371/journal.pone.0089313

2. Sundaramoorthy, R., Fyfe, P. K., andHunter, W. N. (2008) Structure of Staphylococcus aureus EsxA suggests a contribution to virulence by action as a transport chaperone and/or adaptor protein J Mol Biol **383**, 603-614 10.1016/j.jmb.2008.08.047

3. Holm, L. (2020) Using Dali for Protein Structure Comparison Methods Mol Biol **2112**, 29-42 10.1007/978-1-0716-0270-6_3

4. Solomonson, M., Setiaputra, D., Makepeace, K. A. T., Lameignere, E., Petrotchenko, E. V., Conrady, D. G. *et al.* (2015) Structure of EspB from the ESX-1 type VII secretion system and insights into its export mechanism Structure **23**, 571-583 10.1016/j.str.2015.01.002

5. Korotkova, N., Freire, D., Phan, T. H., Ummels, R., Creekmore, C. C., Evans, T. J. *et al.* (2014) Structure of the Mycobacterium tuberculosis type VII secretion system chaperone EspG5 in complex with PE25-PPE41 dimer Mol Microbiol **94**, 367-382 10.1111/mmi.12770

6. Klein, T. A., Shah, P. Y., Gkragkopoulou, P., Grebenc, D. W., Kim, Y., andWhitney, J. C. (2024) Structure of a tripartite protein complex that targets toxins to the type VII secretion system Proc Natl Acad Sci U S A **121**, e2312455121 10.1073/pnas.2312455121

7. Massey, T. H., Mercogliano, C. P., Yates, J., Sherratt, D. J., andLowe, J. (2006) Double-stranded DNA translocation: structure and mechanism of hexameric FtsK Mol Cell **23**, 457-469 10.1016/j.molcel.2006.06.019

8. Olson, A. B., Kent, H., Sibley, C. D., Grinwis, M. E., Mabon, P., Ouellette, C. *et al.* (2013) Phylogenetic relationship and virulence inference of Streptococcus Anginosus Group: curated annotation and whole-genome comparative analysis support distinct species designation BMC Genomics **14**, 895 10.1186/1471-2164-14-895

9. Whitney, J. C., Peterson, S. B., Kim, J., Pazos, M., Verster, A. J., Radey, M. C. *et al.* (2017) A broadly distributed toxin family mediates contact-dependent antagonism between gram-positive bacteria Elife **6**, 10.7554/eLife.26938

10. Aspiras, M. B., Kazmerzak, K. M., Kolenbrander, P. E., McNab, R., Hardegen, N., andJenkinson, H. F. (2000) Expression of green fluorescent protein in Streptococcus gordonii DL1 and its use as a species-specific marker in coadhesion with Streptococcus oralis 34 in saliva-conditioned biofilms in vitro Appl Environ Microbiol **66**, 4074-4083, <https://www.ncbi.nlm.nih.gov/pubmed/10966431>

11. Klein, T. A., Grebenc, D. W., Shah, P. Y., McArthur, O. D., Dickson, B. H., Surette, M. G. *et al.* (2022) Dual Targeting Factors Are Required for LXG Toxin Export by the Bacterial Type VIIb Secretion System mBio e0213722 10.1128/mbio.02137-22
